# Combination Therapy with Oseltamivir and Favipiravir Delays Mortality but Does Not Prevent Oseltamivir Resistance in Immunodeficient Mice Infected with Pandemic A(H1N1) Influenza Virus

**DOI:** 10.3390/v10110610

**Published:** 2018-11-03

**Authors:** Mariana Baz, Julie Carbonneau, Chantal Rhéaume, Marie-Hélène Cavanagh, Guy Boivin

**Affiliations:** Research Center in Infectious Diseases of the CHU of Québec and Laval University, 2705, Boul. Laurier (RC-709), Québec City, QC G1V 4G2, Canada; julie.carbonneau@crchudequebec.ulaval.ca (J.C.); rheaumec@hotmail.com (C.R.); Marie-Helene.Cavanagh@crchudequebec.ulaval.ca (M.-H.C.)

**Keywords:** pandemic influenza virus, immunosuppression, combination therapy, oseltamivir, favipiravir, resistance, mice

## Abstract

Immunosuppressed individuals can shed influenza virus for prolonged periods of time, leading to the frequent emergence of antiviral resistance. We evaluated the benefits of oseltamivir and favipiravir combination therapy compared to single antiviral agents and monitored the emergence of drug-resistant variants in a pharmacologically immunosuppressed mouse model infected with the A(H1N1) pandemic influenza virus. C57BL/6 mice were immunosuppressed with cyclophosphamide and infected with a lethal dose of pandemic influenza A(H1N1) virus. Forty-eight hours post-infection, mice were treated with oseltamivir (20 mg/kg), favipiravir (20 or 50 mg/kg) or both agents BID for 5 or 10 days. Body weight losses, survival rates, lung viral titers, cytokine levels and emergence of resistant viruses were evaluated. Treatment of immunosuppressed mice with high (50 mg/kg) but not low (20 mg/kg) doses of favipiravir in combination with oseltamivir (20 mg/kg) significantly delayed mortality and reduced lung viral titers compared to treatment with a single drug regimen with oseltamivir but did not prevent the emergence of oseltamivir-resistant H275Y neuraminidase variants. Combination therapy with oseltamivir and favipiravir should be considered for evaluation in clinical trials.

## 1. Introduction

Influenza viruses are a leading cause of respiratory infections in both immunocompetent (IC) and immunosuppressed (IS) individuals. In the latter population, influenza is associated with a higher risk of comorbidity, death, as well as prolonged viral shedding [1,2,3].

Currently, antiviral mono-therapy provides suboptimal results in patients who are immunosuppressed [4]. Neuraminidase inhibitors (NAIs) such as oseltamivir, zanamivir, and peramivir are active against influenza A and B viruses and currently constitute the main class of antivirals used for the control of influenza virus infections in both IC and IS individuals. However, the emergence of oseltamivir-resistant viruses has been observed in both prophylactic and treatment settings, particularly in IS patients with prolonged viral shedding [5,6,7,8]. Oseltamivir-resistant viruses of the H1N1 subtype carry most commonly a histidine-to-tyrosine substitution at residue 275 (H275Y) (N1 numbering) in the neuraminidase (NA) protein and this substitution confers cross-resistance to peramivir but not to zanamivir [9].

There is a tremendous need of new antivirals for the treatment of influenza virus infections, particularly in IS individuals. Favipiravir (6-fluoro-3-hydroxy-2-pyrazinecarboxamide), originally known as T-705, is a novel antiviral compound that selectively and potently inhibits the RNA-dependent RNA polymerase (RdRp) of influenza viruses [10], as well as that of many other RNA viruses [11]. Favipiravir is converted to 4-ribofuranosyl-5-triphosphate metabolite by intracellular enzymes and may be misincorporated into the nascent RNA strand as natural ATP and GTP during viral RNA synthesis [12,13]. Favipiravir inhibits influenza A and B viruses, including NAI-resistant variants, both in vitro and in vivo [10,11,14].

Combination therapy is considered one of the best strategies for addressing the issue of antiviral resistance in IS patients. The use of two or more antiviral drugs that target different viral proteins during the viral replication cycle has been shown to reduce viral replication in vitro [15], but few studies have examined this strategy with favipiravir and oseltamivir in an IS mouse model [16].

In this study, we compared the efficacy of oseltamivir, favipiravir and the combination against infection with a recombinant A(H1N1) pandemic influenza virus (A(H1N1)pmd09) [17] in IS mice treated with cyclophosphamide (CP) [18,19,20]. The immunosuppression protocol with CP used here was previously established for influenza virus infection [20] and causes morbidity and mortality with a low dose of virus. The same CP treatment schedule was previously shown to be markedly suppressive on cellular immune factors with an almost total inhibition of natural killer cell response as well as T-and B-cell proliferation [21]. In addition, CP is also a strong suppressor of antibody production [22].

## 2. Materials and Methods

### 2.1. Virus and Cells

The recombinant (rec) influenza A(H1N1)pdm09 wild-type (WT) was generated from the first A(H1N1)pdm09 virus isolated in Québec City (A/Québec/144147/09, GenBank accession number FN434457) using bidirectional pLLBA and pLLBG plasmids as previously described [17]. Madin–Darby Canine Kidney cells overexpressing the α2.6 sialic acid receptor (ST6-GalI-MDCK) cells were kindly provided by Y. Kawaoka from the University of Wisconsin, Madison, WI [23].

### 2.2. Antiviral Compounds

For in vitro studies, oseltamivir carboxylate (the active form of oseltamivir) was synthesized by Hoffmann-La Roche (Basel, Switzerland) and, for in vivo studies, the prodrug oseltamivir phosphate (Tamiflu) was purchased in a local pharmacy. Both compounds were suspended in sterile water. For in vitro studies, favipiravir was purchased from Adooq Bioscience (Irvine, CA, USA) and diluted in DMSO to a stock concentration of 100 mM. For in vivo studies, this compound was purchased from BOC Sciences (Shirley, NY, USA) and prepared in sterile water supplemented with 74.6 mg/mL of meglumine excipient.

### 2.3. Animals

Animal experiments were approved by the Animal Care Ethics Committee of Université Laval and mice were used in accordance to the guidelines of Canadian Council on Animal Care. Six-to eight-week-old female C57BL/6 mice were purchased from Charles River Canada (St-Constant, QC, Canada). Animals were housed four to five per cage, kept under conditions which prevented cage-to-cage infections and fed with sterilized food and water.

### 2.4. Immunosuppression Regimen

Mice were treated intraperitoneally (i.p.) with 100 mg/kg of cyclophosphamide (CP; Sigma, St-Louis. MO, USA) every 4 days starting one day before infection [20].

### 2.5. Experimental Design

#### 2.5.1. Pandemic A(H1N1) Infection in IS Mice

Groups of five mice were treated i.p. with 100 mg/kg of CP 24 h before intranasal (i.n.) inoculation with 10^2^ to 10^6^ TCID_50_ of rec A(H1N1)pdm09 in 30 µL of PBS, and again on days +3, +7, and +11 post-infection (p.i.). Mice were observed daily for 14 days for clinical signs of illness, including weight loss, ruffled fur, and hunching. Mice were humanely euthanized by cervical dislocation under isoflurane anesthesia if they lost 20% of their original body weight.

#### 2.5.2. Immunosuppression Regimen

The immunosuppression efficacy of the CP regimen was confirmed by observing the development of antibodies in the CP treated (IS) and non-treated (IC) group of mice. Five mice were treated i.p. with 100 mg/kg of CP 24 h before intranasal (i.n.) inoculation with 10^2^ TCID_50_ of rec A(H1N1)pdm09 in 30 µL of PBS, and again on days +3, +7 and +11 post-infection (p.i.). Five non-CP treated mice were infected as described above. All mice were observed daily for 14 days for clinical signs of illness, including weight loss, ruffled fur, and hunching. Mice were humanely euthanized by cervical dislocation under isoflurane anesthesia if they lost 20% of their original body weight. The level of specific antibodies was determined by microneutralization (MN) assay [24] on days 0 (before infection) and 11 p.i. for the IS mice and days 0, 11, 14 and 21 for IC mice. Briefly, serial twofold dilutions of heat-inactivated serum were prepared starting from a 1:20 dilution. Equal volumes and virus were mixed and incubated for 60 min at room temperature. The residual infectivity of the virus-serum mixture was determined in ST6-GalI-MDCK cells using four wells for each dilution of serum. Neutralizing antibody (NtAb) titer was defined as the reciprocal of the serum dilution that completely neutralized the infectivity of 100 TCID_50_ of the virus as determined by the absence of cytopathic effect on ST6-GalI-MDCK cells at day 4.

#### 2.5.3. Antiviral Studies with Oseltamivir, Favipiravir, or the Combination

Groups of eight mice were treated (IS mice) or not (IC mice) with CP on days −1, +3, +7, +11, +15 and +19 and infected on day 0 with 10^2^ TCID_50_ of rec A(H1N1)pdm09 in 30 µL of PBS. To mimic clinical conditions, treatments were initiated 48 h p.i. (by gavage) with 20 mg/kg of oseltamivir phosphate, 20 mg/kg of favipiravir or the combination delivered as a single solution BID for 5 days (experiment 1), 20 mg/kg of oseltamivir phosphate, 20 mg/kg of favipiravir and the combination BID for 10 days (experiment 2) or 20 mg/kg of oseltamivir phosphate, 50 mg/kg of favipiravir or the combination BID for 10 days (experiment 3). Control animals inoculated with virus were treated with meglumine for 5 or 10 days. Animals were weighed daily for 20 (experiment 1) or 21 (experiments 2 and 3) days and monitored for clinical signs and mortality. For experiments 2 and 3, four mice per group were sacrificed on days 8, 12, and 15 p.i., and the lungs were removed aseptically. For determination of the viral titers, harvested lung tissues were homogenized in 1 mL of Dulbecco’s modified Eagle medium (Life Technologies Corporation, Burlington, ON, Canada) containing 2× antibiotic-antimycotic solution (penicillin. streptomycin and amphotericin B) (Invitrogen-Gibco) using Omni Tip^TM^ Homogenizer. Tissue homogenates were clarified by centrifugation (2000× *g* for 5 min) and supernatants were collected and titrated on ST6-GalI-MDCK cells monolayers. The infectivity was determined by recording the presence of cytopathic effect (CPE) and titers were expressed as log_10_ TCID_50_/mL of tissue [25].

### 2.6. Cytokine and Chemokine Analysis

The concentration of 23 cytokines and chemokines (IL-1α, IL-1β, IL-2, IL-3, IL-4, IL-5, IL-6, IL-9, IL-10, IL-12(p40), IL-12(p70), IL-13, IL-17A, eotaxin, G-CSF, GM-CSF, IFN-γ, KC, MCP-1, MIP-1α, MIP-1β, RANTES and TNF-α) on day 12 p.i. were measured in lung homogenates (*n* = 3/group) using the Bio-Plex Pro^TM^ Mouse Cytokine 23-plex panel (Bio-Rad Laboratories, Mississauga, ON, Canada) according to the manufacturer’s instructions. Cytokine and chemokine concentrations were expressed as pg/mL of lung.

### 2.7. Reverse-Transcription Droplet Digital PCR (RT-ddPCR)

Viral RNA was extracted from 100 µL of lung homogenates using the MagNA Pure LC total nucleic acid isolation kit (Roche Molecular System, Laval, QC, Canada) and eluted in 100 µL of elution buffer. The ddPCR workflow and data analyses were performed with the One-Step RT-ddPCR Advanced Supermix (Bio-Rad Laboratories, Mississauga, ON, Canada) according to the manufacturer’s instructions and as previously described [26]. The limit of detection for this assay is 1.4 copies per well.

### 2.8. RT-PCR Amplification and Sanger Sequencing

RNA was isolated from lung homogenates as described above. cDNA was synthesized using random hexamer primers (Amersham Pharmacia Biotech, Piscataway, NY, USA) and the SuperScript II reverse transcriptase enzyme (Life Technologies Corporation). Full-length viral NA and PB1 cDNAs were amplified by PCR using the Phusion high-fidelity DNA polymerase (New England BioLabs, Whitby, ON, Canada) and specific primers (available upon request) in standard conditions. The nucleotide sequences of the PCR products were determined using the ABI 3730 DNA analyzer, and chromatogram peaks were analyzed using BioEdit, version 7.0.5 (Carlsbad, CA, USA).

### 2.9. Susceptibility Assays

The phenotype of resistance to oseltamivir carboxylate was determined by a fluorometric NA inhibition assay as described elsewhere [27]. Stock viruses (rec H1N1pdm09 WT or H275Y used as controls) and viruses isolated from mouse lungs on days 8, 12, and 15 p.i. (previously passaged once in ST6-GalI-MDCK cells) were standardized to an NA activity level 10-fold higher than that of 2’-(4-methylumbelliferyl)-α-d-*N*-acetylneuraminic acid (MUNANA; Sigma, St-Louis, MO, USA) substrate (final concentration of 100 µM). Favipiravir susceptibility was determined from mouse lungs harvested on day 15 p.i. by conventional plaque reduction assay (PRA) [28].

### 2.10. Statistical Analyses

Lung viral titers and cytokine/chemokine levels were compared by one-way analysis of variance (ANOVA) with Tukey’s multiple-comparison post-test whereas mean days of death were compared using Student’s *t* test. A Log-Rank (Mantel–Cox) test was used to compare Kaplan–Meier survival plots. All analyses were done using GraphPad, version 7 (La Jolla, CA, USA).

## 3. Results

### 3.1. Pandemic A(H1N1) Infection in IS Mice

**Figure 1:**
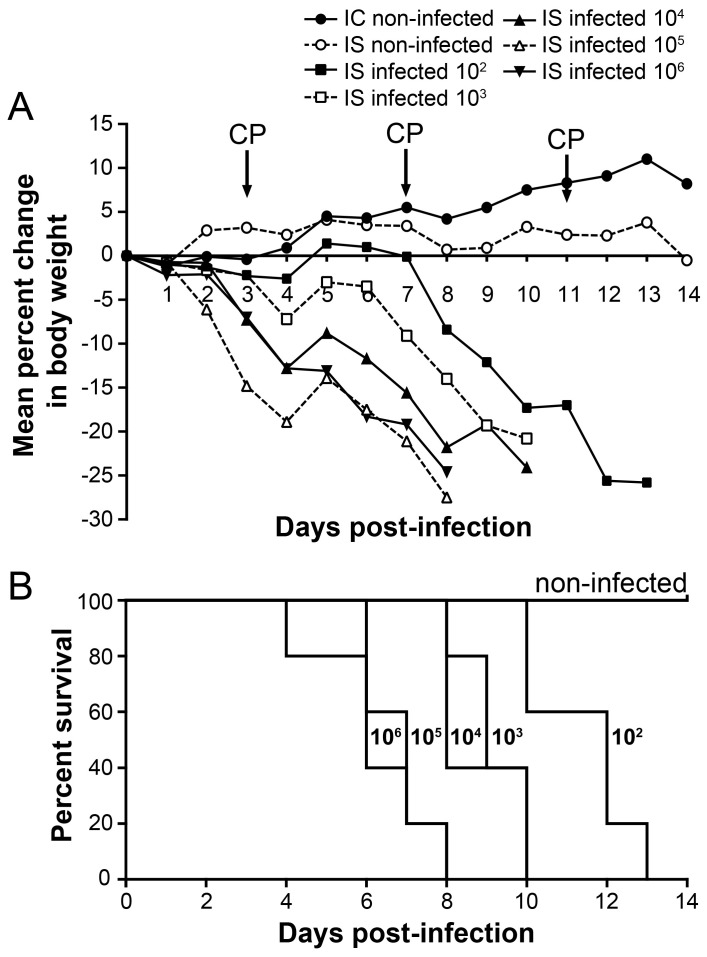
Weight loss (**A**) and survival rate (**B**) of IS C57BL/6 mice intranasally infected with 10^2^ to 10^6^ TCID_50_ of rec A(H1N1)pdm09 in 30 µL of PBS. Animals were monitored daily for 14 days for clinical signs of illness, including weight loss, ruffled fur, and hunching. Mice were sacrificed when they lost 20% of their original body weight.

In order to validate our IS mouse model and to evaluate the virulence of the rec A(H1N1) pdm09 virus, we inoculated groups of five IS mice with serial 10-fold dilutions (10^2^ to 10^6^) of the virus and observed them for 14 days for clinical signs of illness (weight loses, ruffled fur, and hunching). Weight loss was observed in all groups of infected IS mice starting as early as day 1 p.i. Inoculation with 10^2^ to 10^6^ TCID_50_ of rec A(H1N1) pdm09 resulted in significant, dose-dependent weight loss that rapidly progressed to ≥20% of body weight (MLD_50_ < 10^2^ TCID_50_) (Figure 1A,B). Mean days of death (MDD) of mice infected with 10^2^, 10^3^, 10^4^, 10^5^, and 10^6^ TCID_50_ of rec A(H1N1) pdm09 were 11.4, 9.2, 8.8, 6.4, and 6.6, respectively (data not shown). In contrast, the same recombinant virus has a MLD_50_ of approximately 10^5^ TCID_50_ in IC mice (data not shown). We chose an inoculum of 10^2^ TCID_50_ for all subsequent studies because it induces 100% mortality in IS mice with animals dying between days 10 and 13.

### 3.2. Immunosuppression Regimen

As expected, all mice treated with CP and inoculated with a low dose of 10^2^ TCID_50_ of rec A(H1N1) pdm09 died on day 11 p.i., and none of them developed antibodies at this time point, confirming the effectiveness of the CP treatment (data not shown). Conversely, IC mice (not treated with CP) infected with the same low dose of rec A(H1N1) pdm09 did not show mortality nor morbidity (data not shown) and elicited antibody titers with a geometric mean titer (GMT) of 153 on day 11 p.i., which increased to 1140 and 1708 by day 14 and 21 p.i., respectively.

### 3.3. Five-Day Single and Combined Therapies in IS and IC Mice

**Figure 2:**
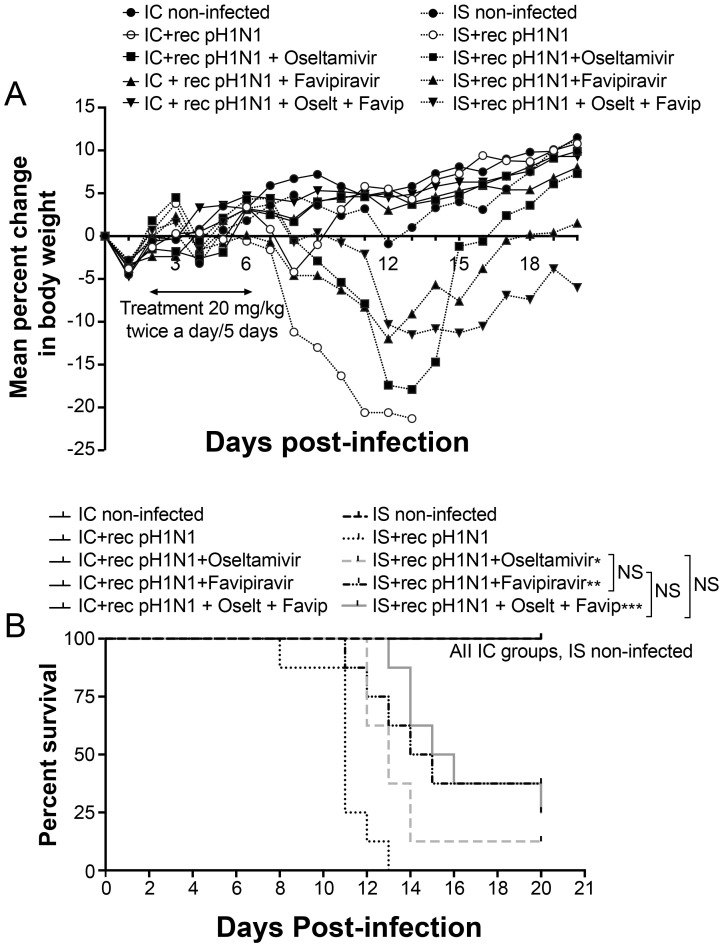
Evaluation of five-day single and combined therapy in IS and IC mice. C57BL/6 mice intranasally infected with 10^2^ TCID_50_ of rec A(H1N1)pdm09 received meglumine (placebo), 20 mg/kg of oseltamivir, favipiravir or the combination by gavage BID for five days, starting at 48 h post-infection (p.i.). An uninfected group (meglumine) was added as control. (**A**) Animals were monitored daily for 20 days for clinical signs of illness, including weight loss, ruffled fur, and hunching and sacrificed when they lost 20% of their original body weight. (**B**) Kaplan–Meier survival curves for IS mice were compared using the Log-Rank (Mantel–Cox) test (* *p* < 0.05, ** *p* < 0.01 and *** *p* < 0.001).

To assess the therapeutic efficacy of oseltamivir, favipiravir or the drug combination, we first treated IS and IC mice with 20 mg/kg of oseltamivir phosphate, 20 mg/kg of favipiravir or the combination, twice a day (BID) for 5 days (experiment 1). Weight loss and mortality were monitored for 20 days. As expected, all infected IC mice survived (MDD > 20) and increased their body weight between 5 to 10% of their initial weight (day 0) by day 20 p.i. (Figure 2A). In contrast, all placebo-treated infected IS mice (control group) died by day 13 p.i (MDD = 11) (data not shown). IS mice treated with any drug regimen did not show significant body weight loss while receiving treatment (i.e. from day 2 to 6 p.i.), but all groups started to lose weight one day after treatment was completed (day 7 p.i.). The survival rates of IS mice treated with 20 mg/kg of oseltamivir, favipiravir or the drug combination for 5 days were 12.5% (1/8; *p* < 0.05). 37.5% (3/8; *p* < 0.01), and 25% (2/8; *p* < 0.001), respectively, compared to the non-treated group (0%) (Figure 2B). Statistically significant increases in MDD were seen by comparing the groups treated with oseltamivir, favipiravir, and the combination to the control group with MDD values of 13 (*p* < 0.05), 13 (*p* < 0.05), 15.3 (*p* < 0.01) and 11, respectively. The combination treatment significantly increased (*p* < 0.05) the MDD of mice compared to that of mice that received oseltamivir but not favipiravir (*p >* 0.05) (data not shown). Thus, although the combination therapy did not protect IS mice from death, it did delay mortality when administrated at a concentration of 20 mg/kg of each drug, twice daily for 5 days.

### 3.4. Ten-Day Single and Combined Therapies with Low Dose of Favipiravir in IS Mice

**Figure 3:**
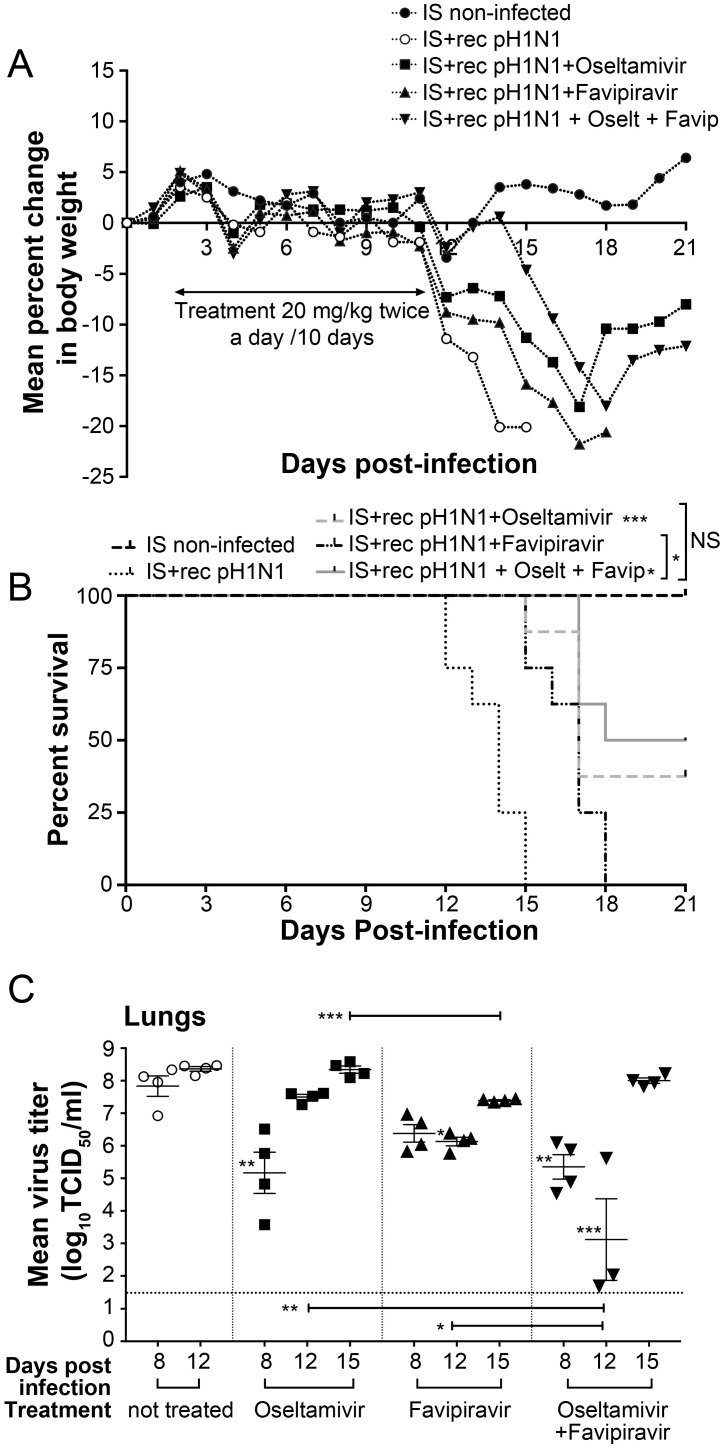
Evaluation of ten-day single and combined therapy with low dose of favipiravir in IS mice. C57BL/6 mice intranasally infected with 10^2^ TCID_50_ of rec A(H1N1)pdm09 received meglumine (placebo), 20 mg/kg of oseltamivir, favipiravir or the combination by gavage BID for ten days, starting at 48 h post-infection (p.i.). An uninfected group (meglumine) was added as control. (**A**) Animals were monitored daily for 21 days for clinical signs of illness, including weight loss, ruffled fur, and hunching and sacrificed when they lost 20% of their original body weight. (**B**) Kaplan–Meier survival curves for IS mice were compared using the Log-Rank (Mantel–Cox) test (* *p* < 0.05, and *** *p* < 0.001). (**C**) Lung viral titers ± standard error of mean were determined by TCID_50_ using ST6-GalI-MDCK cells for groups of four mice euthanized on days 8, 12, and 15 post-infection and compared by one-way analysis of variance (ANOVA) with Tukey’s multiple-comparison post-test (* *p* < 0.05, ** *p* < 0.01 and *** *p* < 0.001). The dotted horizontal line indicates the lower limit of detection; i.e. 10^1,5^ TCID_50_/mL.

In order to evaluate if a prolonged period of treatment would decrease morbidity and mortality in IS treated mice, we infected IS mice as described above and treated them with the same drug regimen but continuing treatment for 10 days (experiment 2). Weight loss and mortality were monitored for 21 days. All placebo-treated infected IS mice died between days 12 and 15 p.i (MDD = 13.6). Similar to experiment 1, all treated groups started to lose weight one day after completion of treatment (day 12 p.i.) (Figure 3A). The survival rates for IS mice treated with 20 mg/kg of oseltamivir, favipiravir or the combination were 37.5% (3/8; *p* < 0.05), 0% (0/8; *p* > 0.05) and 50% (4/8; *p* < 0.001), respectively (Figure 3B). Statistically significant increases in MDD were seen by comparing the groups treated with the oseltamivir, favipiravir and the combinations to the control group, with MDD values of 16.6 (*p* < 0.001), 16.6 (*p* < 0.001), 17.2 (*p* < 0.001) and 13.6, respectively. However, the combination treatment did not significantly increase the MDD of mice compared to that of mice that received monotherapy (*p >* 0.05) (data not shown).

On day 8 p.i., the lung viral titers (LVT) of IS mice treated with oseltamivir or the oseltamivir-favipiravir combination were statistically lower than those of non-treated mice, with mean titers of 10^5.2^ (*p* < 0.01), 10^4.9^ (*p* < 0.01) and 10^7.8^ TCD_50_/mL, respectively (Figure 3C). On day 12 p.i., one day after the end of the treatments, mice treated with favipiravir or oseltamivir-favipiravir combination had significantly lower LVTs than those of non-treated mice, with mean titers of 10^6.1^ (*p* < 0.05), 10^3.1^ (*p* < 0.001) and 10^8.4^ TCD_50_/mL, respectively. However, after discontinuation of the drug, surviving IS mice had a rapid rebound in viral replication and LVTs on day 15 p.i. were higher than previous time points for all treated groups (Figure 3C).

Interestingly, RT-ddPCR assay designed for the detection and quantification of H275Y associated with oseltamivir-resistant variant demonstrated that two out of four mice treated with oseltamivir contained low or intermediate levels of mutant population on days 8 (2.26 and 1.92%) and 12 p.i. (20.09 and 1.96%) (Table 1). However, on day 15 p.i. we only detected 5.45% of mutant population in one mouse. Mice treated with the oseltamivir-favipiravir combination had similar % of mutant population on days 8 and 12 p.i. but, on day 15 p.i., they showed higher proportion of H275Y variant with three mice having 19.1, 16.91 and 26.24% of H275Y variant (Table 1).

Phenotypic studies did not reveal resistance by NAI assay with mean IC_50_ value similar to that of non-treated mice (Table 1). Sequence analysis of the NA gene from the lungs collected on day 15 p.i. did not show the presence of the H275Y in mice treated with oseltamivir. However, the chromatogram of the NA genes from three mice treated with the combination and positive for H275Y by ddPCR showed a mixed population at this codon. No other mutation was found in the NA genes of mice. To examine favipiravir susceptibility, we determined the IC_50_ values of viruses isolated on day 15 p.i. by PRA. Mean IC_50_ values on day 15 p.i. for mice treated with favipiravir or oseltamivir-favipiravir combination were 12 and 12.3 μM compared to 10.9 μM for non-treated mice (Table 1). Sequence analysis of the PB1 genes from the lungs collected on day 15 p.i. showed no amino acid changes compared to the non-treated group.

### 3.5. Ten-Day Single and Combined Therapies with High Dose of Favipiravir in IS Mice

**Figure 4:**
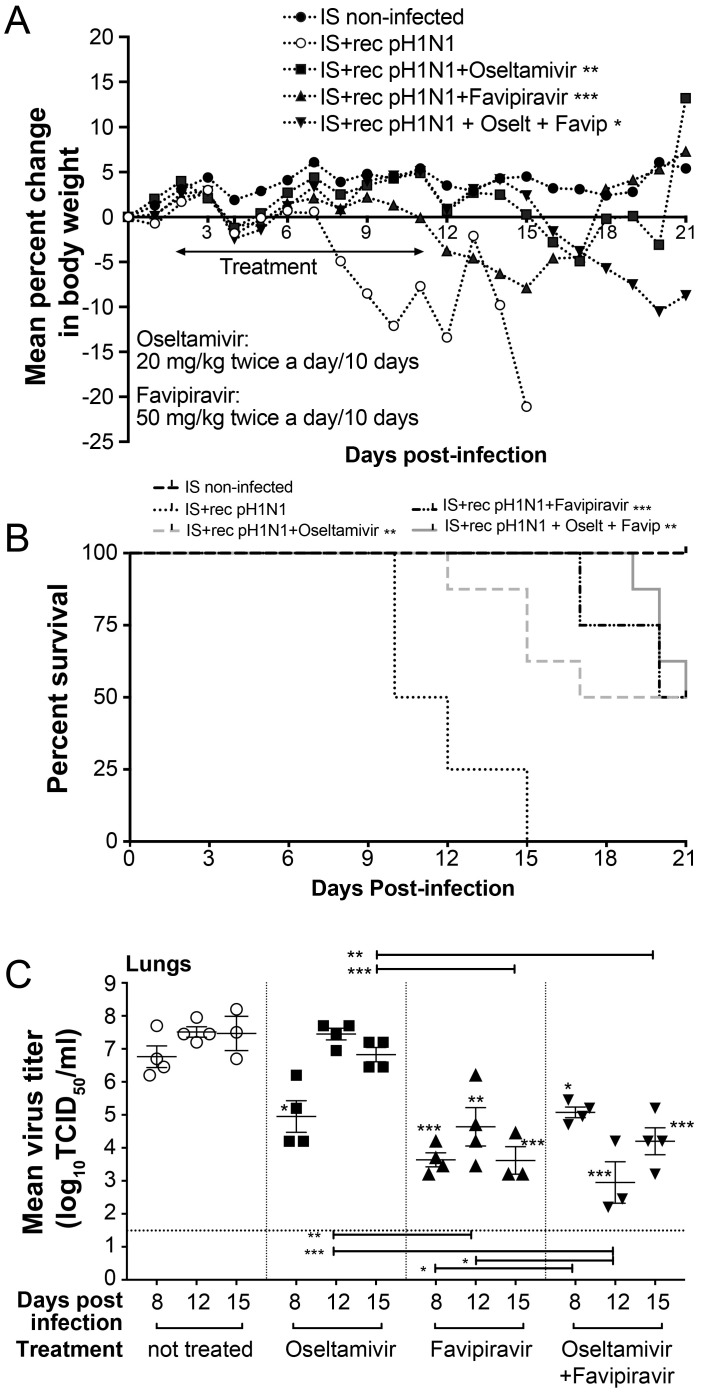
Evaluation of ten-day single and combined therapy with high dose of favipiravir in IS mice. C57BL/6 mice intranasally infected with 10^2^ TCID_50_ of rec A(H1N1)pdm09 received meglumine (placebo), 20 mg/kg of oseltamivir, 50 mg/kg of favipiravir or the combination by gavage BID for ten days, starting at 48 h post-infection (p.i.). An uninfected group (meglumine) was added as a control. (**A**) Animals were monitored daily for 21 days for clinical signs of illness, including weight loss, ruffled fur, and hunching and sacrificed when they lost 20% of their original body weight. (**B**) Kaplan–Meier survival curves for IS mice were compared using the Log-Rank (Mantel–Cox) test (** *p* < 0.01 and *** *p* < 0.001). (**C**) Lung viral titers ± standard error of mean were determined by TCID_50_ using ST6-GalI-MDCK cells for groups of four mice euthanized on days 8, 12 and 15 post-infection and compared by one-way analysis of variance (ANOVA) with Tukey’s multiple-comparison post-test (* *p* < 0.05, ** *p* < 0.01 and *** *p* < 0.001). The dotted horizontal line indicates the lower limit of detection, 10^1,5^ TCID_50_/mL.

We then evaluated if a higher concentration of favipiravir (50 mg/kg BID for 10 days) would prevent morbidity and emergence of oseltamivir resistance in IS treated mice. IS mice were infected as described above and treated with 20 mg/kg of oseltamivir phosphate, 50 mg/kg of favipiravir or the combination BID for 10 days (experiment 3). Weight loss and mortality were monitored for 21 days. All control IS mice died between day 10 and 15 p.i (MDD = 11.5). Body weight losses over a 21-day period were reduced in the oseltamivir-, favipiravir-, or oseltamivir-favipiravir-groups (Figure 4A). Survival rates of IS mice treated with 20 mg/kg of oseltamivir, 50 mg/kg of favipiravir or the combination for 10 days were 50% for all groups with *p* values <0.05, <0.01 and <0.001, respectively, compared to control (Figure 4B). Statistically significant increases in MDD were seen by comparing the groups treated with the oseltamivir, favipiravir and the combination to the control group with MDD values of 14.7 (*p* < 0.05), 18 (*p* < 0.001), 20 (*p* < 0.001) and 11.5, respectively. In addition, the combination treatment significantly increased the MDD of mice compared to that of mice that received monotherapy with a *p* value <0.01 for mice treated with oseltamivir and <0.05 for those treated with favipiravir (data not shown).

LVTs of mice treated with favipiravir or the oseltamivir-favipiravir combination were statistically lower than those of non-treated and oseltamivir-treated mice at all time points (Figure 4C), highlighting the benefit of administrating higher doses of favipiravir. On day 12 p.i., LVTs of mice treated with the combination therapy were significantly lower than those of favipiravir-treated mice.

Interestingly, RT-ddPCR assay demonstrated that the group treated with oseltamivir-favipiravir combination had higher % of H275Y mutant population than that of mice treated with oseltamivir monotherapy on days 8 and 12 p.i. and, on day 15 p.i., three mice out of four had 16.4, 6.5 and 1.7% of H275Y variant (Table 2). The oseltamivir resistance phenotype associated with H275Y was not detected by NAI assay (Table 2). Sequence analysis of the NA gene from the lungs collected on day 15 p.i. showed a mixed population at codon 275 in only one mouse treated with the combination. No other mutation was found in the NA genes of mice. Favipiravir susceptibility of influenza viruses recovered on day 15 p.i. did not show a resistant phenotype by PRA. Sequence analysis of the PB1 gene from mice treated with favipiravir or the combination showed no amino acid changes compared to the non-treated group.

**Figure 5:**
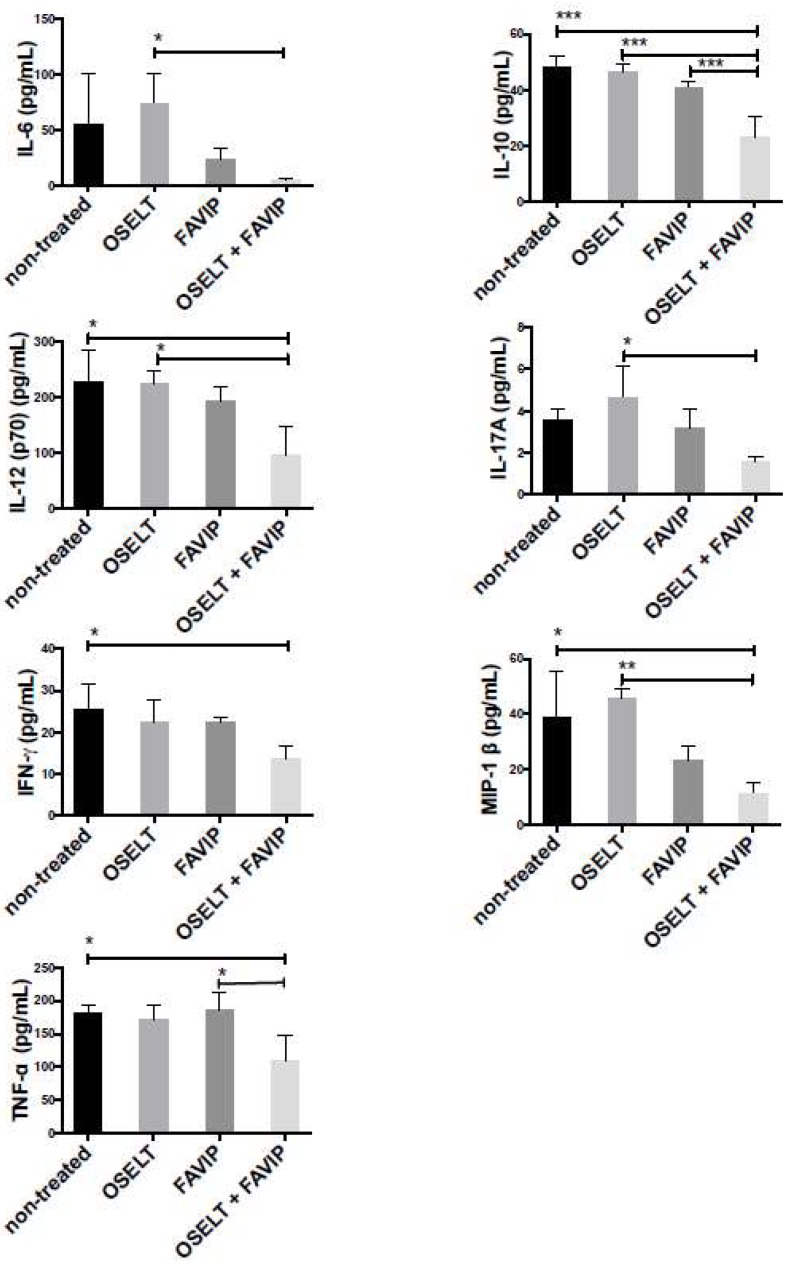
Pulmonary cytokine/chemokine levels on day 12 post-infection in IS C57BL/6 mice intranasally infected with 10^2^ TCID_50_ of rec A(H1N1)pdm09. Mice received meglumine (non-treated), 20 mg/kg of oseltamivir, 50 mg/kg of favipiravir or the combination by gavage BID for ten days, starting at 48 h post-infection (p.i.). Bars represent mean values ± SDs (*n* = 3/group). One-way analysis of variance (ANOVA) with Tukey’s multiple-comparison post-test (* *p* < 0.05 and ** *p* < 0.01) was used for the comparisons.

Oseltamivir and favipiravir combination significantly reduced pulmonary levels of IL-10, IL-12(p70), IFN-γ, MIP-1α and TNF-α cytokines, compared to non-treated mice on day 12 p.i. (Figure 5). Similarly, the combination decreased IL-6, IL-10, IL-12(p70), IL-17A and MIP-1β levels compared to oseltamivir-treated mice as well as IL-10 and TNF-α levels compared to favipiravir-treated mice (Figure 5). No changes in the levels of the other cytokines were observed between all groups of mice (data not shown).

## 4. Discussion

Immunosuppressed individuals are highly susceptible to influenza virus infections, leading to prolonged virus shedding, longer hospitalizations, and usually more severe complications. The two major options for controlling influenza infections, vaccines and antivirals, have several limitations in this population. In addition, prolonged virus shedding also increases the risk of emergence of drug-resistant variants to oseltamivir, the most widely used NAI [5,29,30,31]. Therapeutic approaches with antiviral combinations are not well established and limited information is available from studies in hospitalized or severely ill patients with influenza virus infections. A recent phase 2 trial of a combination of oseltamivir, amantadine, and ribavirin showed a significant decrease in viral shedding compared to oseltamivir monotherapy, but the difference was not associated with improved clinical benefit [32]. So far, no combinations of proven value are currently recommended for the treatment of influenza in IS populations. The combination of multiple agents can potentially provide therapeutic benefits such as additive or synergistic inhibition of viral replication, less toxicity (by reducing doses) and limited resistance rates compared with monotherapy.

Treatment of IS mice with 20 mg/kg of oseltamivir, favipiravir or the combination BID for 10 days was able to delay, although not completely preventing, mortality nor the emergence of oseltamivir-resistant variants in the animals, with very high titers of virus detected in the lungs on day 15 p.i. (four days after the end of the treatment). Interestingly, repeated doses of higher concentrations of favipiravir (50 mg/kg/BID) or the oseltamivir-favipiravir combination decreased mortality by 50% compared to the non-treated group. In addition, lung titers of mice were significantly lower than those of non-treated or oseltamivir-treated mice at all time points p.i. However, low levels of oseltamivir-resistant viruses were still detected in the viral mixture by the ultra-sensitive RT-ddPCR method. Interestingly, the NA inhibitor-resistant viruses were not detected by initial screening using an NA-inhibition assay, probably due to the lower sensitivity of this technique and because virus propagation in cell culture in the absence of the drug may have altered the ratio of drug-resistant and wild-type variants. No substitutions were detected in the polymerase PB1 gene of the viruses recovered from the favipiravir-treated mice on day 15 p.i. by standard sequencing and this correlated with a susceptible phenotype by plaque reduction assay.

In infected IC young adults, nasal wash levels of IL-6, TNF-α and IFN-γ were reduced by oseltamivir treatment [33]. In our IS mouse study, these cytokines were not reduced after oseltamivir treatment; however, levels of IL-6 and TNF-α were reduced when mice were treated with the combination of oseltamivir and favipiravir, which correlates with a reduction in LVTs.

Favipiravir (AVIGAN®) has been only approved in Japan for influenza pandemic preparedness in 2014. A recent study conducted in Japan examined the antiviral susceptibility of 57 pairs of influenza viruses isolated from nasal swabs from adult patients positive for influenza in phase III clinical trials [34]. Although it is still unclear what is the best favipiravir dose to treat infected humans, patients from this study were treated for one or two days with 1200/400 mg and 400 mg BID the following day. Amino acid substitutions in the RdRp subunits (PB1, PB2 and PA) were found after 2 days of favipiravir but no virus with statistically significant reduced susceptibility was detected. In our study, none of the viruses isolated after 10 days of favipiravir treatment displayed reduced susceptibility to this drug, highlighting the potential of this compound even under immunosuppressive conditions. A recent study by Kiso et al., showed similar results (i.e. increased survival times, prolonged viral replication, emergence of resistance to oseltamivir but not to favipiravir), in nude mice infected with A(H1N1)pdm09 even when treatment (25 mg/kg of oseltamivir and 30 mg/kg of favipiravir once a day for 28 days) was started one hour p.i. [16]. However, this treatment did not prevent mortality. In our study, we chose a 48-h delay before initiating treatment (to mimic a more realistic clinical infection) with 20 mg/kg of oseltamivir and higher concentrations of favipiravir (50 mg/kg) BID for 10 days resulting in 50% survival rate at 21 days. Thus, favipiravir dosage is an important parameter in establishing combination therapy protocols.

Collectively, we found that the virus replicated for long periods of time in our IS mouse model similar to studies done in IS individuals [5,35], severe combined immunodeficient (SCID) mice [36] and IS ferrets [37]. Notably, oral administration of high concentrations of favipiravir or the oseltamivir-favipiravir combination not only increased survival rates and delayed mortality but also significantly decreased lung virus titers. However, such a combination was not completely satisfactory with rapid rebound in viral titers shortly after the end of therapy and emergence of oseltamivir-resistant variants. Additional combination regimens should be evaluated in IS models including increased favipiravir doses and drugs with different mechanisms of action such as PB2 and PA inhibitors currently under development [38].

## Figures and Tables

**Table 1:** Oseltamivir and favipiravir susceptibilities of viruses recovered from individual lungs of IS mice infected with the pH1N1 influenza virus and treated with oseltamivir (20 mg/kg). favipiravir (20 mg/kg) or the combination for 10 days.

		NAI Assay (Oseltamivir)		PRA Assay (Favipiravir)		ddPCR (Mut 275Y)	
Mouse #	Days of Treatment	IC50 (nM)	Mean IC50 (nM)	IC50 (µM)	Mean IC50 (µM)	%	Mean %
**Not treated**							
1	12	0.3	0.3 ± 0.1	13.8	10.9 ± 1.7	0.0	0.0 ± 0.0
2		0.2		10.5		0.0	
3		0.3		9.5		0.0	
4		0.4		9.9		0.0	
**Oseltamivir 20 mg/kg/BID**							
1	8	0.5	0.6 ± 0.0	ND	ND	0.0	1.1 ± 1.0
2		0.6		ND		2.3	
3		0.6		ND		0.1	
4		0.6		ND		1.9	
1	12	0.4	0.5 ± 0.0	ND	ND	0.5	5.8 ± 8.3
2		0.4		ND		0.5	
3		0.5		ND		20.1	
4		0.5		ND		2.0	
1	15	0.4	0.3 ± 0.1	ND	ND	5.5	1.8 ± 2.2
2		0.2		ND		0.3	
3		0.2		ND		0.1	
4		0.4		ND		1.4	
**Favipiravir 20 mg/kg/BID**							
1	8	NA	NA	ND	ND	NA	NA
2		NA		ND		NA	
3		NA		ND		NA	
4		NA		ND		NA	
1	12	NA	NA	ND	ND	NA	ND
2		NA		ND		NA	
3		NA		ND		NA	
4		ND		ND		ND	
1	15	NA	NA	12.8	12.0 ± 1.0	NA	NA
2		NA		11.5		NA	
3		NA		10.6		NA	
4		NA		13		NA	
**Oseltamivir 20 mg/kg + Favipiravir 20 mg/kg/BID**							
1	8	0.5	0.6 ± 0.1	ND	ND	0.4	1.1 ± 1.0
2		0.4		ND		0.1	
3		0.6		ND		1.2	
4		0.8		ND		2.7	
1	12	0.7	0.6 ± 0.1	ND	ND	7.3	2.9 ± 3.2
2		0.5		ND		1.4	
3		0.6		ND		0.00	
4		ND*		ND*		ND*	
1	15	1.1	0.8 ± 0.3	11.9	12.3 ± 1.4	19.1	15.6 ± 9.6
2		0.8		10.3		16.9	
3		0.3		13		0.1	
4		0.8		14		26.3	

Abbreviations: NAI: Neuraminidase inhibition assay; PRA: Plaque reduction assay; ddPCR: Droplet digital PCR; IC_50_: 50% inhibitory concentration; NA: Not applicable; ND: Not done; * Dead mouse.

**Table 2:** Oseltamivir and favipiravir susceptibilities of viruses recovered from individual lungs of IS mice infected with the pH1N1 influenza virus and treated with oseltamivir (20 mg/kg), favipiravir (50 mg/kg) or the combination for 10 days.

		NAI Assay (Oseltamivir)		PRA Assay (Favipiravir)		ddPCR (Mut 275Y)	
Mouse #	Days of Treatment	IC50 (nM)	Mean IC50 (nM)	IC50 (µM)	Mean IC50 (µM)	%	Mean %
**Not treated**							
1	15	0.3	0.4 ± 0.1	11.5	10.3 ± 1.3	0.0	0.0 ± 0.0
2		0.5		8.5		0.0	
3		0.3		10.9		0.0	
4		ND*		ND *		ND *	
**Oseltamivir 20 mg/kg/BID**							
1	8	0.5	0.6 ± 0.1	ND	ND	5.0	3.2 ± 3.2
2		0.4		ND		7.6	
3		0.7		ND		0.0	
4		0.6		ND		0.4	
1	12	0.5	0.6 ± 0.1	ND	ND	1.3	1.9 ± 2.0
2		0.7		ND		5.4	
3		0.5		ND		0.7	
4		0.7		ND		0.3	
1	15	0.4	0.5 ± 0.1	ND	ND	0.3	0.1 ± 0.1
2		0.4		ND		0.0	
3		0.6		ND		0.2	
4		0.6		ND		0.0	
**Favipiravir 50 mg/kg/BID**							
1	8	NA	NA	ND	ND	NA	NA
2		NA		ND		NA	
3		NA		ND		NA	
4		NA		ND		NA	
1	12	NA	NA	ND	ND	NA	NA
2		NA		ND		NA	
3		NA		ND		NA	
4		NA		ND		NA	
1	15	NA	NA	13.1	12.3 ± 0.7	NA	NA
2		NA		12.4		NA	
3		NA		11.5		NA	
4		ND*		ND *		ND *	
**Oseltamivir 20 mg/kg + Favipiravir 50 mg/kg/BID**							
1	8	0.7	0.6 ± 0.1	ND	ND	0.1	7.0 ± 6.7
2		0.5		ND		16.1	
3		0.6		ND		1.1	
4		0.5		ND		10.7	
1	12	0.7	0.7 ± 0.1	ND	ND	2.7	5.6 ± 3.2
2		ND*		ND *		ND *	
3		0.6		ND		4.1	
4		0.7		ND		10.1	
1	15	0.9	0.8 ± 0.1	12.6	11.0 ± 1.2	1.0	6.4 ± 6.1
2		0.9		11.6		16.4	
3		0.7		10.4		6.5	
4		0.8		9.5		1.7	

Abbreviations: NAI: Neuraminidase inhibition assay; PRA: Plaque reduction assay; ddPCR: Droplet digital PCR; IC_50_: 50% inhibitory concentration; NA: Not applicable; ND: Not done;* Dead mouse.
